# Physically-constrained evapotranspiration models with machine learning parameterization outperform pure machine learning: Critical role of domain knowledge

**DOI:** 10.1371/journal.pone.0328798

**Published:** 2025-07-23

**Authors:** Yeonuk Kim, Monica Garcia, T. Andrew Black, Mark S. Johnson

**Affiliations:** 1 Institute for Resources, Environment and Sustainability, University of British Columbia, Vancouver, Canada; 2 Division of Hydrologic Sciences, Desert Research Institute, Las Vegas, Nevada, United States of America; 3 Estación Experimental de Zonas Áridas. Consejo Superior de Investigaciones Científicas (EEZA-CSIC), Almería, Spain; 4 Faculty of Land and Food Systems, University of British Columbia, Vancouver, Canada; 5 Department of Earth, Ocean and Atmospheric Sciences, University of British Columbia, Vancouver, Canada; Swedish Meteorological and Hydrological Institute, SWEDEN

## Abstract

Physics-informed machine learning techniques have emerged to tackle challenges inherent in pure machine learning (ML) approaches. One such technique, the hybrid approach, has been introduced to estimate terrestrial evapotranspiration (ET), a crucial variable linking water, energy, and carbon cycles. A key advantage of these hybrid ET models is their improved performance, particularly under extreme conditions, compared to ET estimates relying solely on ML. However, the mechanisms driving their improved performance are not well understood. To address this gap, we developed six hybrid approaches based on different physical formulations of ET and compared them with a pure ML model. All models employed the random forest algorithm and were trained on daily-scale ET observations, in-situ meteorological data and satellite remote sensing. We found a strong correlation (r = 0.93) between the sensitivity of ET estimates to machine-learned parameters and model error (root-mean-square error; RMSE), indicating that reduced sensitivity minimizes error propagation and improves performance. Notably, the most accurate hybrid model (RMSE = 17.8 W m^-2^ in energy unit) utilized a novel empirical parameter, which is relatively stable due to land-atmosphere equilibrium, outperforming both the pure ML model and hybrid models requiring conventional parameters (e.g., surface conductance). These results imply that conventional parameterizations may require reevaluated to effectively integrate physical models with machine learning, as conventional choices may not be optimal for this new, hybrid, paradigm. This study underscores the critical role of domain knowledge in setting up hybrid models, potentially guiding future hybrid model developments beyond ET estimation.

## 1 Introduction

Terrestrial evapotranspiration (ET), which represents the sum of plant transpiration and evaporation from soil and intercepted water, plays a crucial role in Earth’s climate as it is a nexus of the water, energy, and carbon cycles [1–3]. However, direct measurements of ET are limited in space and time, making satellite remote sensing-based ET estimation an essential tool. The remote sensing-based ET models generally fall into two broad categories: physically-based approaches [4–9] and those utilizing machine learning algorithms (ML) [10–14].

In the last decade, ML algorithms have emerged as a popular approach to estimate ET. For example, random forests, support vector machines, and artificial neural networks are most widely applied to directly estimate ET using satellite and meteorological data as inputs [15,16]. More recently, advanced models such as deep learning (e.g., long short-term memory networks) [17] and ensemble frameworks combining multiple ML algorithms have been introduced in ET estimation [18]. These ML models have been shown to estimate ET with high accuracy, especially with the availability of a large number of satellite and field observations, making them a key tool in ET studies.

Despite their advantages, ML-based approaches face several challenges. These include their ‘black-box’ nature, which obscures the internal logic of the models, a reliance on large datasets, and a lack of physical constraints (e.g., surface energy balance and diffusion laws) that can hinder their generalizability and extrapolation capability, especially relevant in the context of climatic change [19,20]. To overcome these limitations, various forms of physics-informed ML, including hybrid modeling, have emerged as promising alternatives [19,21]. Here, the term hybrid approaches specifically refers to models that employ an ML approach as a sub-model to predict intermediate quantities within a physically-based framework [21]. For ET estimation, hybrid models typically employ ML to estimate difficult-to-measure parameters, such as the ratio of actual ET to potential ET (PET) or surface conductance representing soil and vegetation water stress, and then integrate these ML-determined parameters into physically-based ET models [22–30]. These hybrid ET models show promise as they can combine the advantages of both physically-based models and machine learning approaches. Reflecting this trend, for example, the widely used Global Land Evaporation Amsterdam Model (GLEAM) has transitioned from a fully physically based model to a hybrid approach that constrains PET in its latest version [30].

Recent studies have demonstrated that hybrid ET models outperform pure ML models under extreme conditions [22,23,27], but evidence suggests that this advantage is not as pronounced under normal conditions [22,26,27,29]. Importantly, the mechanisms by which hybrid ET models enhance performance over pure ML models remain poorly understood, although it is generally believed that incorporating physical knowledge can lead to model improvements. The underlying assumption is that error propagation from ML-determined intermediate parameters to ET in hybrid approaches may be smaller than the direct ET estimation error using a pure ML model, thereby enhancing the accuracy of ET estimates. However, this assumption holds only if physically-based ET models are not overly sensitive to small variations in these intermediate empirical parameters. To our knowledge, no previous study has explicitly tested this underlying assumption.

To fill this knowledge gap, we examine the extent to which hybrid models enhance ET estimation performance over pure ML models, with a focus on model sensitivity to empirical parameters. We hypothesize that a lower sensitivity of ET estimates to ML-determined empirical parameters leads to improvement in model performance. To test this hypothesis, we have developed six hybrid ET models that incorporate various physical equations and empirical parameters to assess how the physical structure of the model and the choice of empirical parameter influence performance relative to pure ML models.

These hybrid models employ equations based on the Penman equation [31] (one model), the Penman-Monteith equation [32] (two models, one of which is the FAO reference crop ET equation [33]), the Priestley-Taylor equation [34] (one model), and a modified version of the Penman-Monteith equation that includes relative humidity gradients (PM_RH_) [35] (two models). The hybrid ET models employ the random forest (RF) algorithm to estimate intermediate parameters (e.g., the ratio between actual ET and PET, or direct physical quantities such as surface resistance and vertical relative humidity flux). Remote sensing data from the Soil Moisture Active Passive (SMAP) and MODerate resolution Imaging Spectrometer (MODIS) satellite platforms, fused with meteorological observations were used as inputs to the RF algorithm. The machine-learned intermediate parameters were then substituted into each physical equation listed above. We evaluate these hybrid ET models in comparison to daily-scale ET observations derived from eddy-covariance (EC) measurements at 40 AmeriFlux sites across various land cover types and climates.

## 2 Data

In this study, we utilized a combination of satellite and field observations. The satellite data were retrieved using Google Earth Engine [36] while field observations were accessed through the AmeriFlux website (https://ameriflux.lbl.gov/data/download-data).

We utilized surface soil moisture (0−5 cm) data from the SMAP enhanced L3 radiometer version 5 (SPL3SMP_E), which has a spatial resolution of 9 km with 2−3 days revisit time [37]. To ensure high-quality measurements, we specifically used descending orbit measurements. This soil moisture (SM) dataset combines the SMAP L-band radiometer and Sentinel-1 C-band radar to improve the resolution of the data. In addition, we utilized vegetation water content (VegWC) data provided by the SPL3SMP_E product as ancillary data. We also included the fraction of photosynthetically active radiation (FPAR) from the MODIS product MCD15A3H version 6.1 [38] as another satellite input. This product provides FPAR measurements at a spatial resolution of 400 m, and is a 4-day composite. The missing days between satellite data were interpolated using the next available value.

For meteorological and ET observations, we used EC flux tower data. To ensure the use of high-quality EC observations that have undergone standardized processing, including quality control and gap-filling, we employed the AmeriFlux FLUXNET dataset. This dataset was developed using the ONEFlux processing method, which is consistent with the FLUXNET2015 dataset [39]. We obtained meteorological data and daily energy balance-corrected latent and sensible heat fluxes using the Bowen-ratio conservation method [40]. This approach was employed because the physically-based models used in this study require energy balance closure. Here, the latent heat flux (*LE*) derived using the EC systems represents ET observations.

**Fig 1:**
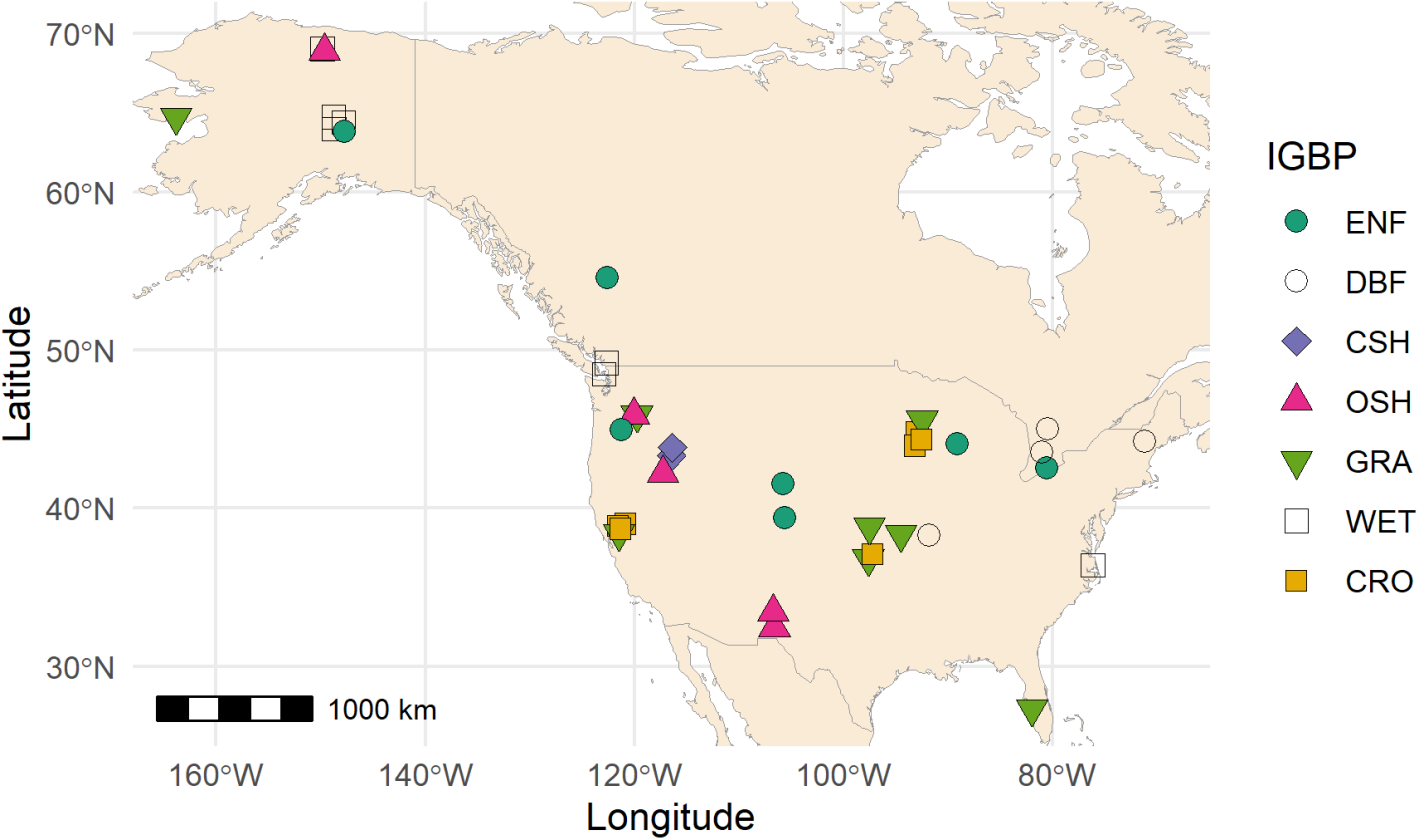
Spatial distribution of 40 AmeriFlux FLUXNET sites used in this study. Each point indicate site locations and the different shapes representing the International Geosphere-Biosphere Programme (IGBP) land cover classification, which include evergreen needleleaf forests (ENF; 7 sites), deciduous broadleaf forests (DBF; 4 sites), closed shrublands (CSH; 2 sites), opened shrublands (OSH; 5 sites), grasslands (GRA; 8 sites), permanent wetlands (WET; 7 sites), and croplands (CRO; 7 sites). Map background from Natural Earth (http://www.naturalearthdata.com).

We selected field data for periods when all required variables were available. Moreover, we only included data for periods for which the quality control flag indicated that more than 80% of the high quality half-hourly data were used to generate the daily ET data. As the SMAP observations were available only after March 2015, we only included field data that overlapped with the satellite observation period. After applying these filtering criteria, we were left with 40 AmeriFlux FLUXNET sites (6 Canadian sites and 34 US sites) representing 39,000 daily observations (Fig 1 and Table 1). The 40 sites span a wide range of land cover types.

**Table 1:** Information for the 40 eddy-covariance sites. The fourth column shows the land cover types based on the International Geosphere-Biosphere Programme (IGBP) classification, which include evergreen needleleaf forests (ENF; 7 sites), deciduous broadleaf forests (DBF; 4 sites), closed shrublands (CSH; 2 sites), opened shrublands (OSH; 5 sites), grasslands (GRA; 8 sites), permanent wetlands (WET; 7 sites), and croplands (CRO; 7 sites). The fifth column shows the Köppen climate classification, which include mid-latitude steppe and desert (Bsh; 2 sites), tropical steppe (Bsk; 3 sites), subtropical steppe (Bwk; 2 sites), humid subtropical (Cfa; 7 sites), hot-summer mediterranean (Csa; 5 sites), warm-summer mediterranean (Csb; 3 sites), hot-summer humid continental (Dfa; 5 sites), warm-summer humid continental (Dfb; 4 sites), subarctic (Dfc; 2), extremely-cold subarctic (Dfd; 4), and Tundra (ET; 2).

Site	Latitude (N)	Longitude (W)	IGBP land cover	Köppen climate class	Period	PI/ Citation
CA-Cbo	44.3167	−79.9333	DBF	Dfb	2015-2020	Staebler [41]
CA-DB2	49.119	−122.995	WET	Csb	2020	Knox [42]
CA-DBB	49.1293	−122.985	WET	Csb	2016-2020	Christen and Knox [43]
CA-LP1	55.1119	−122.841	ENF	Csa	2015-2020	Black [44]
CA-TP3	42.7068	−80.3483	ENF	Dfb	2015-2017	Arain [45]
CA-TPD	42.6353	−80.5577	DBF	Dfb	2015-2017	Arain [46]
US-A32	36.8193	−97.8198	GRA	Cfa	2015-2017	Billesbach et al. [47]
US-ARM	36.6058	−97.4888	CRO	Cfa	2016-2020	Biraud et al. [48]
US-Bi1	38.0992	−121.499	CRO	Csa	2016-2021	Rey-Sanchez et al. [49]
US-Bi2	38.1091	−121.535	CRO	Csa	2017-2021	Rey-Sanchez et al. [50]
US-BZB	64.6955	−148.321	WET	Dfd	2017-2021	Euskirchen [51]
US-BZF	64.7013	−148.312	WET	Dfd	2017-2021	Euskirchen [52]
US-BZo	64.6936	−148.33	WET	Dfd	2018-2021	Euskirchen [53]
US-BZS	64.6963	−148.324	ENF	Dfd	2015-2021	Euskirchen [54]
US-CS2	44.1467	−89.5002	ENF	Dfa	2021	Desai [55]
US-GLE	41.3665	−106.24	ENF	Dfc	2015-2020	Frank and Massman [56]
US-Hn2	46.6889	−119.464	GRA	Bsk	2018−2018	Liu et al. [57]
US-Hn3	46.6878	−119.461	OSH	Bsk	2017-2018	Liu et al. [58]
US-ICs	68.6058	−149.311	WET	ET	2015-2020	Euskirchen et al. [59]
US-ICt	68.6063	−149.304	OSH	ET	2015-2021	Euskirchen et al. [60]
US-Jo1	32.582	−106.635	OSH	Bwk	2015-2020	Tweedie [61]
US-Jo2	32.5849	−106.603	OSH	Bwk	2016-2020	Vivoni and Perez-Ruiz [62]
US-KFS	39.0561	−95.1907	GRA	Cfa	2015-2018	Brunsell [63]
US-KLS	38.7745	−97.5684	GRA	Cfa	2015-2019	Brunsell [64]
US-Me2	44.4523	−121.557	ENF	Csb	2015-2020	Law [65]
US-MOz	38.7441	−92.2	DBF	Cfa	2015-2019	Wood and Gu [66]
US-NC4	35.7879	−75.9038	WET	Cfa	2015-2021	Noormets [67]
US-NGC	64.8618	−163.7	GRA	ET	2018-2019	Torn and Dengel [68]
US-NR1	40.0329	−105.546	ENF	Dfc	2015-2016	Blanken et al. [69]
US-ONA	27.3836	−81.9509	GRA	Cfa	2016-2020	Silveira [70]
US-Rms	43.0645	−116.749	CSH	Bsh	2015-2020	Flerchinger [71]
US-Ro1	44.7143	−93.0898	CRO	Dfa	2015-2016	Baker et al. [72]
US-Ro4	44.6781	−93.0723	GRA	Dfa	2015-2021	Baker and Griffis [73]
US-Ro5	44.691	−93.0576	CRO	Dfa	2017-2020	Baker and Griffis [74]
US-Ro6	44.6946	−93.0578	CRO	Dfa	2017-2021	Baker and Griffis [75]
US-Rwf	43.1207	−116.723	CSH	Bsh	2015-2020	Flerchinger [76]
US-Rws	43.1675	−116.713	OSH	Bsk	2015-2020	Flerchinger [77]
US-Sne	38.0369	−121.755	GRA	Csa	2016-2020	Shortt et al. [78]
US-Tw3	38.1152	−121.647	CRO	Csa	2015-2018	Chamberlain et al. [79]
US-xBR	44.0639	−71.2873	DBF	Dfb	2017-2021	NEON [80]

## 3 Pure ML and hybrid ET models

### 3.1 Random forest model and inputs

**Fig 2:**
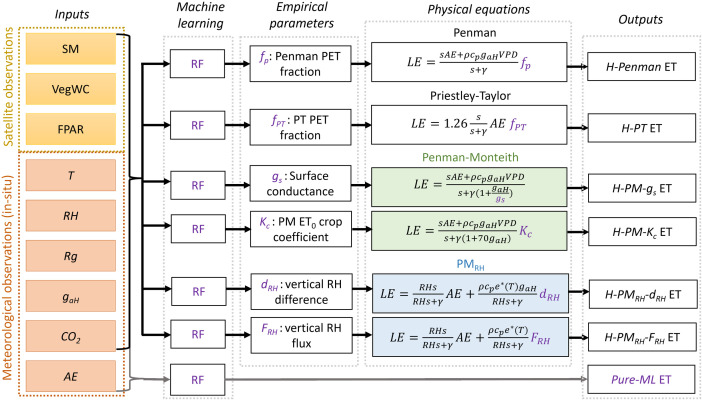
Flow chart of the six hybrid ET models and a pure machine learning model using random forest (RF) algorithms. The model input variables include soil moisture (SM), vegetation water content (VegWC), fraction of photosynthetically active radiation (FPAR), air temperature (T), relative humidity (*RH*), CO_2_ concentration (*CO*_*2*_), aerodynamic conductance (*g*_*aH*_), global radiation (*Rg*), and available energy (*AE*).

We used the RF algorithm to develop one pure ML ET model (*Pure-ML*) and six hybrid ET models (Fig 2). The RF algorithm is an ensemble method of regression trees [81], and involves generating bootstrapped datasets, creating independent regression trees using randomly sampled variables, and aggregating the estimation results of the individual regression trees. RF has been shown to outperform or be comparable to other widely-used machine learning algorithms for estimating ET [15,16].

To create the RF models, we utilized the “Caret” and “randomForest” R packages [82,83], tuning the mtry parameter independently for each model, including both pure ML and hybrid models. Here, the mtry parameter, which is the key hyperparameter in the RF model that requires tuning, indicates the number of randomly sampled variables at each split of a decision tree. We used threefold cross-validation for mtry parameter tuning, initially setting the number of regression trees (ntree) to 100. After identifying the optimal mtry value for each model, we increased ntree to 500 to assess any performance improvement (Table 2). For training and validation, we allocated 75% of total daily ET observations, with the remaining 25% used to test model performance. It should be noted that while the RF algorithm includes several other hyperparameters (e.g., nodesize), mtry and ntree are the most influential in determining model performance and are most commonly tuned during training. Therefore, all other parameters were kept at their default values as defined in the “randomForest” R package.

**Table 2:** Summary of random forest hyperparameters after tuning. Descriptions of the ntree and mtry parameters are provided in the main text. The hybrid models are denoted as follows. H1: *H-Penman*, H2: *H-PT*, H3: *H-PM-g*_*s*_, H4: *H-PM-K*_*c*_, H5: *H-PM*_*RH*_*-d*_*RH*_, and H6: *H-PM*_*RH*_*-F*_*RH*_. Detailed descriptions of each hybrid model are available in Section 3.3.

	Pure RF	RF in H1	RF in H2	RF in H3	RF in H4	RF in H5	RF in H6
ntree	500	500	500	500	500	500	500
mtry	6	3	6	4	3	3	6

We selected the inputs for the ML algorithm based on a similar prior study that evaluated both pure ML and hybrid ET models [22]. Inputs to the RF algorithm included satellite-derived SM, VegWC, FPAR, and in-situ meteorological measurements of air temperature (*T, K*), relative humidity (*RH*), CO_2_ concentration (*CO*_*2*_, μmol mol^-1^), global radiation (*Rg*, W m^-2^), and aerodynamic conductance for sensible heat (*g*_*aH*_, m s^-1^). Here, *g*_*aH*_ was estimated from a semi-empirical model using friction velocity and wind speed [84,85] as described in the following section.

### 3.2 Aerodynamic conductance for heat and water vapour transfer

We estimate the daily-scale aerodynamic conductance for heat (*g*_*aH*_, m s^-1^) by inverting aerodynamic resistance for heat (*r*_*aH*_, s m^-1^). We consider both the aerodynamic resistance to momentum transfer and the additional boundary layer resistance for heat transfer, also known as the excess resistance [84].


$$r_{aH}=\frac{u(z_{r})}{{u_{*}}^{2}}+6.2{u_{*}}^{-0.67}$$ (1)

$$g_{aH}=\frac{1}{r_{aH}}$$ (2)

where *u(z*_*r*_*)* is reference height wind speed (m s^-1^) and u_*_ is friction velocity (m s^-1^). We estimated *r*_*aH*_ (s m^-1^) using the bigleaf R package [85].

We then assume that the boundary layer resistance for water vapour transfer is equivalent to that for sensible heat transfer (i.e., the similarity assumption). Therefore, Equation (2) serves as the aerodynamic conductance for both heat and water vapor. It should be noted that although we utilized the measured friction velocity, *g*_*aH*_ is still an estimated value and not a true observation, as we utilized an empirical estimate of the boundary layer resistance [86]. As a result, the estimated *g*_*aH*_ based on Equations (1) and (2) contains a degree of uncertainty. Addressing the semi-empirical nature of *g*_*aH*_ in developing hybrid ET model is beyond our scope, as it has been explicitly covered in other studies [25,26].

### 3.3 Hybrid ET models

In this study, we developed six distinct hybrid approaches for estimating ET. Each approach incorporates a single intermediate parameter that cannot be directly derived from meteorological data alone. This parameter is estimated using a RF algorithm, which takes both satellite-based and meteorological variables as inputs, as illustrated in Fig 2. The ML–derived intermediate parameter is then integrated into the corresponding physically based equation to compute ET.

To train the RF models, we initially derived empirical parameters by inversely solving each physical model. This derived data then served as the training dataset for the RF algorithm. Occasionally, the derived empirical parameter values were unrealistic, which required constraining derived values within certain limits to enhance model performance (details provided in each model description below). We determined the optimal constraints that minimized error during the training process. For these models, we set the ntree value to 100 and used the default mtry value (number of input variables divided by three), adjusting the constraints accordingly for the training and validation set. The determined optimal limit was then used to tune the mtry parameter, after which the ntree value was increased to 500.

All hybrid ET models used the same input variables for their respective RF algorithms. In contrast, the pure ML model used the same inputs as the hybrid models plus available energy (*AE,* W m^-2^, which is the difference between net radiation and soil heat flux neglecting air-column and above-ground-biomass energy storage), which is not required as an input for the hybrid models because *AE* is incorporated within the physical equations of these models (see Fig 2). This, following the methodology outlined by Zhao et al. [22], ensures a fair and objective comparison between the pure ML and hybrid models. The following subsection provides detailed descriptions of the six hybrid ET models developed in this study.

#### 3.3.1 Hybrid model 1: *H-Penman.*

The first hybrid ET model integrates potential ET (PET) equation with an empirical stress factor predicted by RF algorithm. The Penman equation, also known as open water ET, serves as the upper limit of ET [31]. Some hydrological approaches, such as the Budyko model, estimate ET by reducing the Penman PET [87]. A recent hybrid ET model also employs the Penman equation, predicting ET by multiplying it by a machine learning-determined parameter [27]. *LE* (and thereby ET) can be estimated as follows:


$$LE=[\frac{s}{s+\gamma }AE+\frac{\rho c_{p}}{s+\gamma }g_{aH}VPD]\cdot f_{P}$$ (3)

where $s(=\frac{de^{*}(T)}{dT})$ is the saturation vapour pressure (e*(T)) slope with respect to temperature (*T*) (kPa K^-1^), γ is the psychrometric constant (kPa K^-1^), *AE* is available energy (W m^-2^, which is the difference between net radiation and soil heat flux and heat storage change within above-ground biomass and air-column), *ρ* is the air density (kg m^-3^), *c*_*p*_ is the specific heat of air (J kg^-1^ K^-1^), *VPD* is vapour pressure deficit (kPa), and *f*_*P*_ denotes the empirical parameter reducing Penman Equation for open water to estimate *LE* for the unsaturated land surface.

Theoretically, if there is no bias in *g*_*aH*_, the *f*_*P*_ value should not exceed 1. In our dataset, this holds true for the most part, as 99% of the inferred *f*_*P*_ values from observations are below 1.02. However, a small number of *f*_*P*_ values do exceed this limit. These extreme values posed challenges during the training of the RF model to estimate *f*_*P*_, as they introduced instability and degraded model performance.

To ensure theoretical consistency and improve model performance, we applied a clamping strategy to limit the influence of these outliers. Specifically, we tested upper limits for *f*_*P*_ by systematically varying the clamping threshold from the 99th to the 99.95th percentile in 0.05% increments. Based on this tuning process, we found that setting the upper limit to the 99.85th percentile (1.28) resulted in the lowest RMSE. Accordingly, we constrained *f*_*P*_ values to the range of 0 to 1.28 by replacing any higher values with 1.28. This approach improved the stability and accuracy of the RF model for this hybrid configuration.

#### 3.3.2 Hybrid model 2: *H-PT.*

The second hybrid ET model combines the Priestley-Taylor (PT) equation [34], with a stress factor that is predicted by the RF algorithm. The PT equation also provides PET values under conditions where heat advection is not strong. Satellite-based ET estimation models widely employ the PT PET, and ET is estimated by reducing the PET values through multiplication by stress factors [4,8,88,89]. In this study, we simplify these types of models by using a single stress factor that is estimated using the RF algorithm.


$$LE=1.26\frac{s}{s+\gamma }AE\cdot f_{PT}$$ (4)

where *f*_*PT*_ represents the water stress factor that is predicted by the RF algorithm. Here, 1.26 is the Priestley-Taylor coefficient. Multiplying $1.26\frac{s}{s+\gamma }AE$ by *f*_*PT*_ reduces PT PET to actual *LE*. It should be noted that PT PET implicitly assumes that $1.26\frac{s}{s+\gamma }AE$ represents the upper limit of actual *LE*. However, actual *LE* can sometimes exceed PT PET due to the significant effect of advection and evaporation of intercepted precipitation, especially in irrigated agriculture in dry regions [e.g., 90]. Similar to the first hybrid model, we adjusted the upper limit of *f*_*PT*_ to minimize the RMSE in the validation dataset. We examined the 99% to 99.95% quantile range of inferred *f*_*PT*_ values and determined that setting *f*_*PT*_ between 0 and 2.18 covers 99.45% of the inferred values. This Hybrid-PT model is similar to the approach of Koppa et al. [24], but they are not exactly same in that Koppa et al. [24] partitioned ET into soil evaporation and plant transpiration.

#### 3.3.3 Hybrid model 3: *H-PM-g*_*s*_.

The third hybrid model is based on the Penman-Monteith (PM) equation [32]. The PM equation is also referred to as the big-leaf model because it parameterizes the land surface as a single large leaf, where surface conductance (or inversely, surface resistance) represents water regulation by this big leaf.


$$LE=\frac{sAE+\rho c_{p}g_{aH}VPD}{s+\gamma (1+\frac{g_{aH}}{g_{s}})}$$ (5)

where *g*_*s*_ is the surface conductance (m s^-1^).

In this hybrid approach, *g*_*s*_ is estimated using the RF algorithm with satellite and meteorological inputs. Following Zhao et al. (2019), the RF predicts the logarithmic value of *g*_*s*_ instead of the original scale, as the logarithmic value is more normally distributed. Since the RF model should be trained based on measurements, we infer *g*_*s*_ by inverting Equation (5), and this information is used to train the RF model. In some cases, the inferred *g*_*s*_ values may be physically unrealistic (e.g., negative) due to the uncertainty in measurements and *g*_*aH*_ estimations. In such cases, *g*_*s*_ is set to the minimum value (i.e., 1% quantile of inferred *g*_*s*_ excluding negative *g*_*s*_) if *LE* <= 0, and to the maximum value (99% quantile of inferred *g*_*s*_ excluding negative *g*_*s*_) if *LE* > 0. Similar to previous hybrid models, the upper and lower limits were selected to minimize RMSE in the validation dataset. This approach enables effective training of the RF model while ensuring accurate reproduction of *LE* when *g*_*s*_ is precisely predicted.

The hybrid ET model based on the PM equation with *g*_*s*_ estimation has become a widely used approach [22,23,26,28,29]. It is worth noting that the PM equation linearizes the saturation vapor pressure curve versus temperature (known as the Clausius‐Clapeyron relationship), which can introduce bias when the temperature difference between the land and the atmosphere is large. To resolve this issue, Zhao et al. [22] employed a quadratic form of the PM equation [91], and Chen et al. [23] employed an alternative equation to PM based on the exponential Clausius‐Clapeyron relationship [92]. We also tested the quadratic form of the PM equation and found similar performance to the original PM equation. For the sake of conciseness, we present results only for the original PM equation.

#### 3.3.4 Hybrid model 4: *H-PM-K*_*c*_.

The fourth hybrid model is also based on the PM equation. Specifically, we employ PM based reference ET (ET_0_) approach proposed by FAO [33]. In this approach, *LE* (and thereby ET) can be estimated by multiplication of ET_0_ and crop coefficient (*K*_*c*_):


$$LE=[\frac{sAE+\rho c_{p}g_{aH}VPD}{s+\gamma (1+70g_{aH})}]\cdot K_{c}$$ (6)

Recognizing the challenges in estimating *g*_*s*_, the FAO ET_0_ method sets *g*_*s*_ to 1/70 (m s^-1^), representing well-watered grass. ET_0_ sometimes serves as an upper limit of ET, similar to PET, as ET rarely exceeds ET_0_ values. Consequently, *K*_*c*_ is typically not significantly larger than 1, similar to *f*_*p*_ and *f*_*PT*_. However, some inferred *K*_*c*_ values based on observations significantly exceed theoretical expectations, similar to the cases of *f*_*p*_ and *f*_*PT*_, which can degrade model performance. Therefore, we set the range of *K*_*c*_ from 0 to 1.77, based on the 99.2% quantile of the inferred *K*_*c*_. This upper limit was also determined by tuning to minimize the RMSE of the validation dataset.

#### 3.3.5 Hybrid model 5: *H-PM*_*RH*_*-d*_*RH*_.

The fifth hybrid ET model employs the PM equation expressed using relative humidity (PM_RH_) proposed by Kim et al. [35]. The PM_RH_ ET expression is similar to the PM equation but does not incorporate surface conductance. Instead, ET is expressed as a sum of two terms: “Surface Flux Equilibrium (SFE)” ET and the ET flux driven by the vertical relative humidity difference. The equation is:


$$LE=\frac{RHs}{RHs+\gamma }AE+\frac{\rho c_{p}e^{*}(T)}{RHs+\gamma }g_{aH}d_{RH}$$ (7)

where *RH* is atmosphere relative humidity, and *d*_*RH*_ is the vertical relative humidity difference between the land surface and the atmosphere (i.e., $d_{RH}={RH}_{surf}-RH$, where *RH*_*surf*_ is land surface relative humidity). The first term represents SFE ET, an estimate of ET under equilibrium conditions, which can be easily calculated from *AE* and meteorological observations [93]. The multiplier in the second term, i.e., $\frac{\rho c_{p}e^{*}(T)}{RHs+\gamma }g_{aH}$, can be also estimated from meteorological observations, but *d*_*RH*_ cannot be directly measured. Therefore, we aim to estimate *d*_*RH*_ using the RF algorithm, noting *d*_*RH*_ is influenced by surface water limitations [35]. It should be noted that the original expression for ET by Kim et al. [35] used $e^{*}(T_{surf})$ in the second term, but we approximate it in Equation (7) by using $e^{*}(T)$ as the difference is marginal [35].

Similar to other hybrid approaches, the RF model in the *H-PM*_*RH*_*-d*_*RH*_ model is trained using inferred *d*_*RH*_ from in-situ observations by inverting Equation (7). It should be noted that, unlike other water stress parameters, *d*_*RH*_ can be both positive and negative, depending on whether the land surface is wet or dry relative to atmospheric conditions. The predicted *d*_*RH*_ from the RF model is then used in Equation (7) to estimate ET.

#### 3.3.6 Hybrid model 6: *H-PM*_*RH*_*-F*_*RH*_.

Recognizing semi-empirical nature of *g*_*aH*_, the sixth hybrid model employs a ML algorithm to estimate *g*_*aH*_ *d*_*RH*_ instead of *d*_*RH*_, while using the PM_RH_ equation.


$$LE=\frac{RHs}{RHs+\gamma }AE+\frac{\rho c_{p}e^{*}(T)}{RHs+\gamma }F_{RH}$$ (8)

where $F_{RH}(=g_{aH}d_{RH})$ is relative humidity flux constrained by the vertical difference of relative humidity and aerodynamic conductance.

The target empirical parameter *F*_*RH*_ is estimated using the RF algorithm, trained with inferred *F*_*RH*_ values from measurements. Since *d*_*RH*_ can be both positive and negative, *F*_*RH*_ can also be both positive and negative. The *H-PM*_*RH*_*-F*_*RH*_ model is quite similar to the *H-PM*_*RH*_*-d*_*RH*_ model, but its physical component of the equation is not affected by the uncertainty caused by the semi-empirical *g*_*aH*_ equation.

### 3.4 Model evaluation

The root-mean-square error (RMSE) was used as the primary statistical evaluation metrics. Additionally, we present the ratio of the standard deviation of the modeled values to that of the observations ($\sigma_{m}/\sigma_{o}$) to evaluate how well the model captures the variability of the observations. Accurately capturing the standard deviation is known to be crucial for predicting extreme hydrologic events (e.g., the Kling–Gupta efficiency introduced by Gupta et al. [94]). We also present coefficient of determination (*R*^*2*^) to compare the ML performance in estimating each intermediate parameter for the hybrid models, as RMSE is not directly comparable across parameters due to the differing units of each parameter.

To assess the ability of the models to perform under extreme conditions, such as droughts and heatwaves, we sampled ET values corresponding to the 0th-3th percentiles (i.e., < 3%) and 97th-100th percentiles (i.e., > 97%) of environmental variables for each site using test set results [22,27].

## 4 Derivative-based global sensitivity assessment

We evaluate the sensitivity of each hybrid model to empirical parameters, which are key components in understanding model behavior. The physically-based components of the hybrid ET models are analytically differentiable, enabling the straightforward calculation of first-order sensitivities through partial derivatives:


$$Local\;Sensitivity\;Coefficient=\frac{\partial LE}{\partial x}$$ (9)

where *x* represents the intermediate empirical parameter in each hybrid model: *f*_*P*_*, f*_*PT*_*, K*_*c*_*, g*_*s*_*, d*_*RH*_*,* and *F*_*RH*_.

To compare the impacts of uncertainty in ML-derived empirical parameter (*x*) on the resulting ET estimates, and to standardize these impacts across different units of $\frac{\partial LE}{\partial x}$, a sensitivity index can be used by scaling the local sensitivity coefficients by the characteristic range of variation of each parameter (e.g., standard deviation) [95] to provide a local sensitivity index:


$$Local\;Sensitivity\;Index=\sigma_{x}\frac{\partial LE}{\partial x}$$ (10)

However, our primary interest lies in assessing global sensitivities to compare the impact of each empirical parameter across the different hybrid models. While variance-based methods like Sobol’s are commonly employed, the analytically differentiable nature of our hybrid models’ physical components allows us to use Derivative-based Global Sensitivity Measures (DGSM) [96]. This approach is supported by research demonstrating theoretical links with the well-established Sobol’s method [97]. The DGSM approach uses the following equation:


$$v=E[{\left(\frac{\partial LE}{\partial x}\right)}^{2}]$$ (11)

where *E* denotes expectation, so that *v* is the expectation value of ${\left(\frac{\partial LE}{\partial x}\right)}^{2}$. While DGSM methods typically involve estimating partial derivatives numerically, in this study we utilize analytical solutions obtained by differentiating. This approach not only enhances the precision and reliability of our sensitivity measures but also simplifies implementation.

Analogous to the local sensitivity index, we define the derivative-based global sensitivity index as follows.


$$Global\;Sensitivity\;Index=\sigma_{x}v^{1/2}$$ (12)

This index is particularly useful as it is expressed in the same units as the output ET, revealing the variability of ET due to the variability of the empirical parameters. This facilitates a direct comparison across the empirical parameters, enhancing our understanding of their importance in different hybrid modeling frameworks.

## 5 Results

### 5.1 Tuning hyperparameters vs. employing hybrid approaches

**Fig 3:**
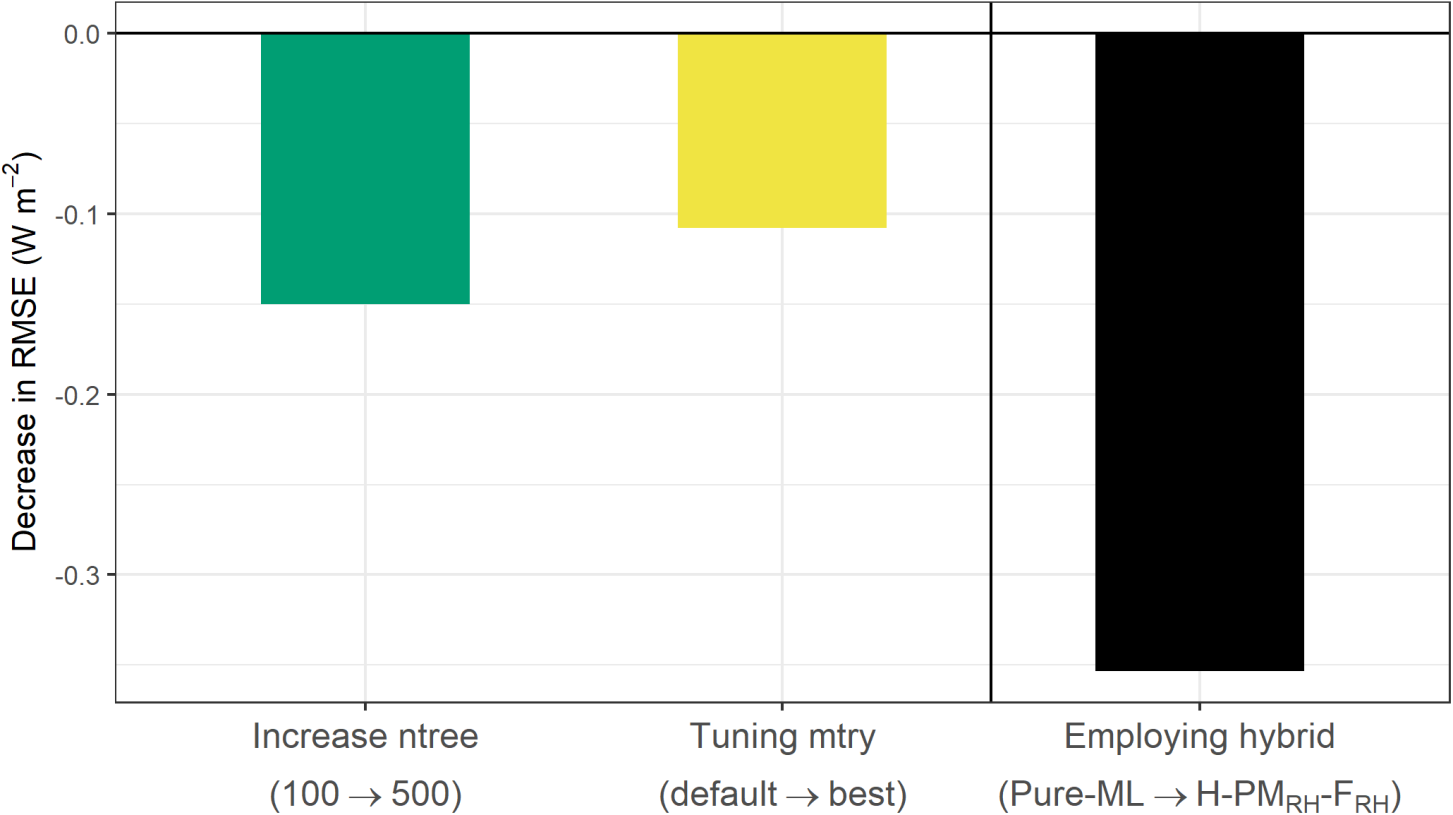
Comparison of model performance changes during the RF hyperparameter optimization of the *Pure-ML* model and the implementation of the best hybrid model. The optimization process includes: (1) increasing ntree from 100 to 500, and (2) tuning mtry from its default value (number of input variables divided by three) to the optimal value. This hyperparameter tuning is then compared with the performance improvement when selecting the best hybrid model, identified as the *H-PM*_*RH*_*-F*_*RH*_ model. These results are based on the training set, with ntree = 100 serving as the baseline setting.

We first examine the impact of employing a hybrid modeling approach on model performance compared to hyperparameter tuning during model training (Fig 3). Starting with the baseline *Pure-ML* model where ntree is set to 100, we observed that increasing ntree to 500 led to a slight improvement in model performance, as indicated by a small reduction in RMSE. Tuning the mtry parameter from its default value (i.e., number of input variables divided by three) to the optimal value resulted in an additional, though modest, decrease in RMSE.

However, when comparing these *Pure-ML* results to the performance of the hybrid models, the difference becomes more significant. The *H-PM*_*RH*_*-F*_*RH*_ model, identified as the best-performing hybrid model, achieved the lowest RMSE in the training/validation dataset. Replacing the *Pure-ML* model with the *H-PM*_*RH*_*-F*_*RH*_ model reduced RMSE by approximately 0.35 W m^−2^. Although the absolute value of this reduction may seem small, it is substantial when compared to the more modest improvements gained from hyperparameter tuning. This comparison underscores that the hybrid approach can provide a greater improvement in model accuracy than hyperparameter tuning.

### 5.2 Test set performance

**Fig 4:**
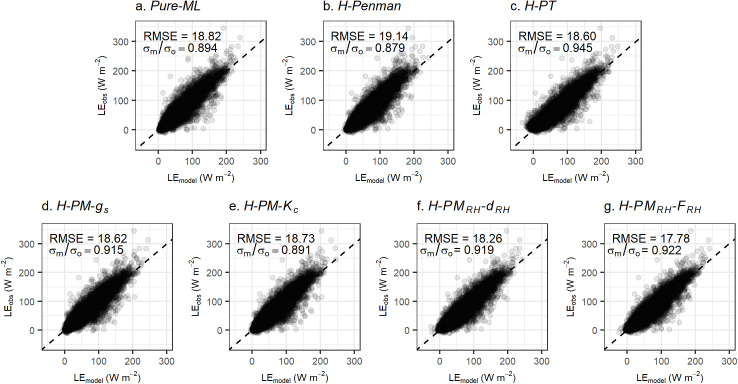
Comparison of test set model performance of *Pure-ML* model (a) and six hybrid models (b-g). the dashed lines represent the one-to-one lines, the y-axis represents energy balance corrected *LE* observations from eddy covariance, while the x-axis shows the model estimated values. The unit of RMSE is in W m^-2^. For reference, 28.46 W m^-2^ of LE for 24 hours is equivalent to 1 mm day^-1^ of ET at 15 °C.

Next, we evaluated the test set performance of the *Pure-ML* model and six hybrid models for estimating *LE* (Fig 4 and Table 3). These test set results were based on ntree set to 500 and the optimal mtry for each model. While the daily *LE* estimation performance of the *Pure-ML* model and the six hybrid models were similar, the hybrid models generally demonstrated better performance in terms of RMSE, with the exception of the *H-Penman* model.

**Table 3:** Summary of test set model performance. The hybrid models are denoted as follows. H1: *H-Penman*, H2: *H-PT*, H3: *H-PM-g*_*s*_, H4: *H-PM-K*_*c*_, H5: *H-PM*_*RH*_*-d*_*RH*_, and H6: *H-PM*_*RH*_*-F*_*RH*_.

	Pure RF	H1	H2	H3	H4	H5	H6
RMSE (W m^-2^)	18.82	19.14	18.60	18.62	18.73	18.26	17.78
$\sigma_{m}/\sigma_{o}$	0.894	0.879	0.945	0.915	0.891	0.919	0.922

Regarding the ratio of standard deviations ($\sigma_{m}/\sigma_{o}$), the *H-PT* model showed the closest value to 1, followed by the *H-PM*_*RH*_*-F*_*RH*_ model. Notably, the *H-PM*_*RH*_*-F*_*RH*_ model demonstrated the most significant improvement compared to the *Pure-ML* model, showing the lowest RMSE among all models, and the second highest value for $\sigma_{m}/\sigma_{o}$. The consistency of the *H-PM*_*RH*_*-F*_*RH*_ model’s lowest RMSE in both the test set and training/validation set evaluations further confirms its superior performance.

**Fig 5:**
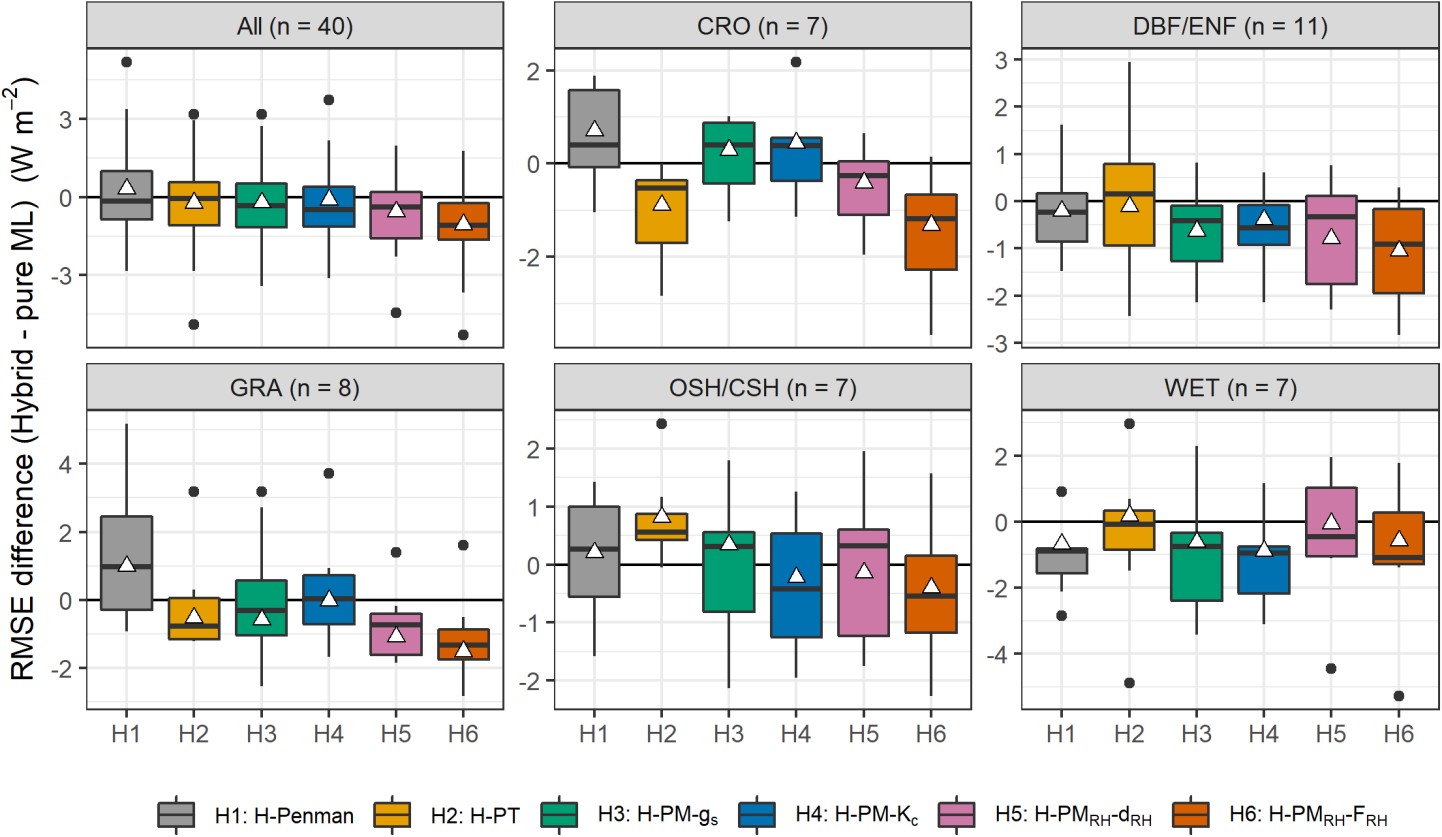
Boxplot comparison of test set RMSE differences (Hybrid minus *Pure-ML*) across the IGBP land cover types: all combined (All), cropland (CRO), deciduous broadleaf and evergreen needleleaf forests (DBF/ENF), grassland (GRA), open shrubland/closed shrubland (OSH/CSH), and wetland (WET). The RMSE difference is shown for each hybrid model relative to the *Pure-ML* model. The number of sites in each land cover type is indicated by *n*. The box plots show the distribution of RMSE differences across individual sites, while the white triangles represent the mean RMSE difference across all sites in each IGBP category, not grouped by each site. Negative values indicate that the hybrid model outperforms the Pure-ML model.

Fig 5 presents a comparison of the test set RMSE differences between the hybrid models and the *Pure-M*L model across different land cover types. The boxplots illustrate the distribution of RMSE differences for each hybrid model across the test sites within each land cover type. The white triangles indicate the mean RMSE difference for each model, offering a summary measure of performance that does not group by individual site variations. A negative RMSE difference indicates that a hybrid model outperforms the *Pure-ML* model, reflecting a reduction in error.

The results show that, on average, the hybrid models tend to outperform the *Pure-ML* model across most land cover types. The *H-PM*_*RH*_*-F*_*RH*_ model consistently shows the largest reductions in RMSE across all land cover types, except for wetlands, and it was the only model to show performance improvement across all IGBP categories. In wetlands, Penman or Penman-Monteith-based models perform better, likely due to the saturated land surface conditions. However, the *H-Penman* model shows higher RMSE differences in land cover types such as CRO and GRA, indicating that it may not perform as effectively in water-limited environments.

### 5.3 Intermediate parameters’ predictability and sensitivity

**Fig 6:**
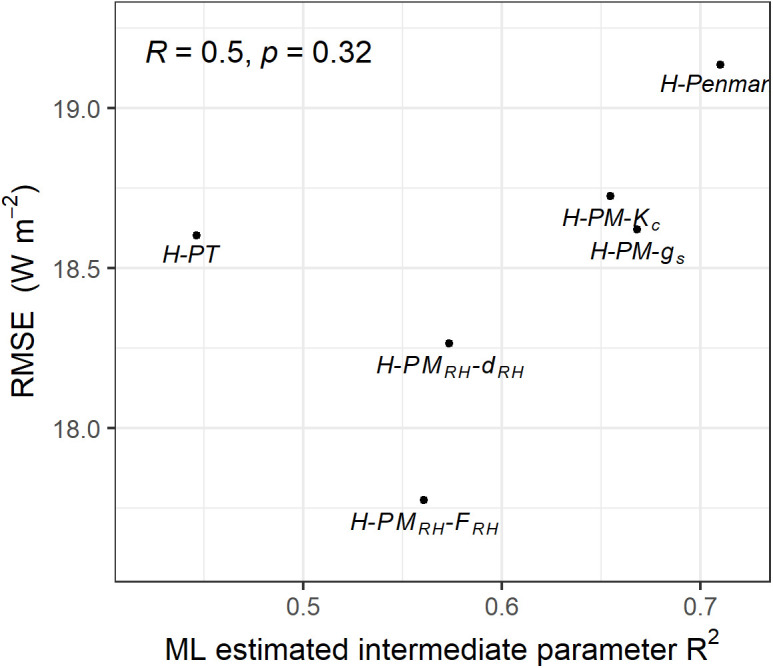
Relationship between the test set RMSE of the hybrid ET models and the test set *R*^2^ of each model’s intermediate parameter estimated by ML. The Pearson’s correlation coefficient (R) between the x and y axes values is presented in the upper-left corner.

The test set performance of the six hybrid ET models reveals varying levels of accuracy across the models. In this section, we explore the underlying reasons for these differences in accuracy. Our first focus is on determining whether better performance of the ML algorithm in estimating each model’s intermediate parameter (i.e., *f*_*P*_*, f*_*PT*_*, K*_*c*_*, g*_*s*_*, d*_*RH*_*,* and *F*_*RH*_) correlates with improved ET estimation (Fig 6). Since the units of the intermediate parameters differ, RMSE is not an appropriate metric. Instead, we use *R*^*2*^ as a more suitable statistical measure for comparing the predictability of these intermediate parameters. Here, *R*^*2*^ is based on the test set results.

Interestingly, the results show that a higher *R*^*2*^ for intermediate parameter estimation does not necessarily correspond to better ET estimation performance. In fact, with the exception of one outlier, the H-PT model, lower *R*^*2*^ values for the intermediate parameters are generally associated with higher ET estimation performance as assessed using RSME values. For instance, the H-Penman model, which has the highest *R*^*2*^ for its intermediate parameter, has the lowest performance in ET estimation (i.e., the highest RMSE). This counterintuitive finding suggests that when ML techniques are used to estimate a parameter that is inherently more difficult to predict, it may enhance the overall accuracy of the final ET prediction. This could be because the ML model is better leveraged when tasked with predicting more complex or less predictable parameters, thus maximizing its capability and impact on the final model performance.

**Fig 7:**
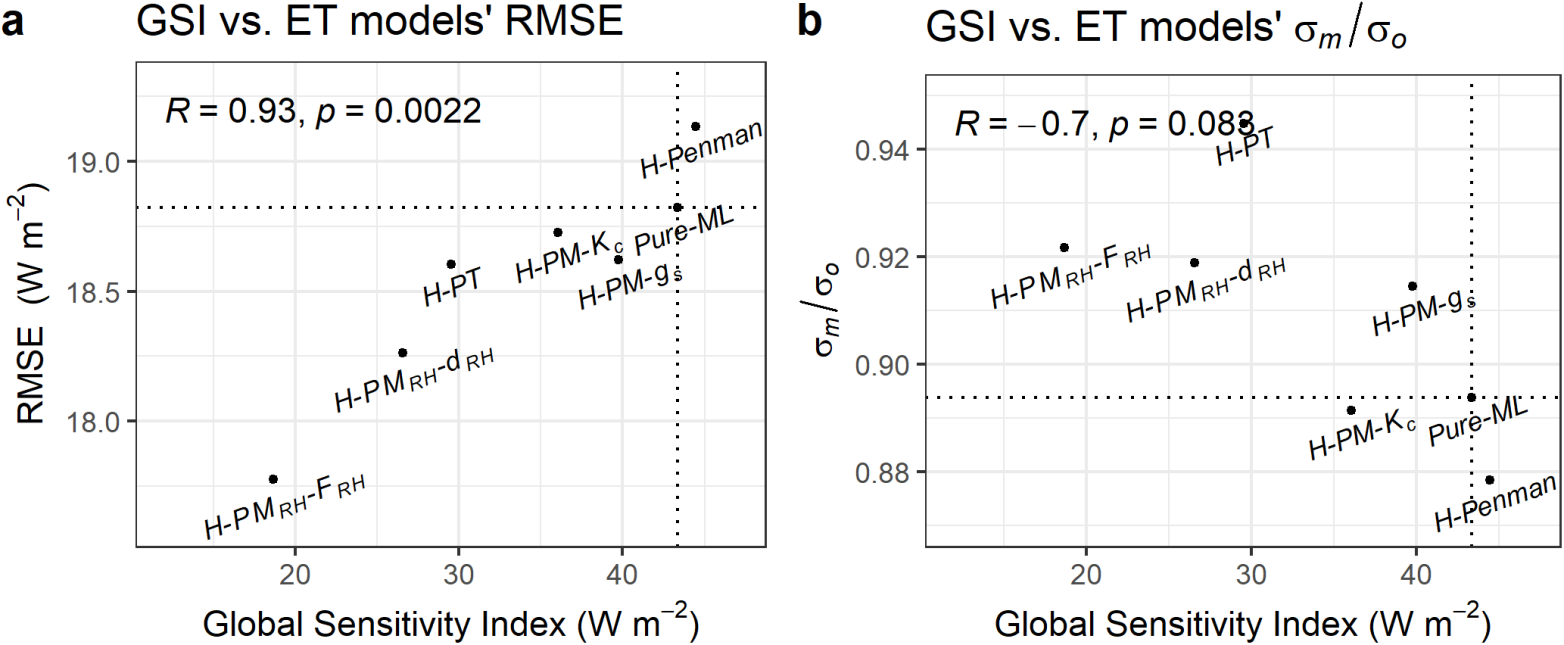
Relationship between the derivative-based Global Sensitivity Index (GSI) and model performance metrics. The x-axis represents GSI and the y-axis represents the models’ performance (test set RMSE) (a) or scaled standard deviation ($\sigma_{m}/\sigma_{o}$) (b). The dotted lines indicate the results of the *Pure-ML* model. The correlation (R) between GSI and the performance metrics are also presented.

Next, we conducted a sensitivity analysis. As we indicated earlier, we hypothesized that the lower sensitivity of ET estimates to ML-determined empirical parameters would lead to improved model performance. To test this hypothesis, we evaluated the relationship between the derivative-based Global Sensitivity Index and the RMSE as shown in Fig 7a. The correlation coefficient (*R* = 0.93*, p < 0.05*) between the Global Sensitivity Index (GSI) and RMSE suggests a strong positive relationship, indicating that models with lower sensitivity to empirical parameters tend to have lower RMSE values. This implies that decreased sensitivity to empirical parameters in hybrid models can contribute to reduced estimation errors, supporting our hypothesis.

The dotted lines in Fig 7a indicate the results of the *Pure-ML* model, serving as a benchmark for comparison. The *H-Penman* model is the only hybrid model whose performance is degraded compared to the *Pure-ML* model, exhibiting a higher RMSE and a higher GSI than the *Pure-ML* model. Among the models, *H-PM*_*RH*_*-F*_*RH*_ exhibited the lowest RMSE and one of the lowest GSI values, indicating its superior performance likely driven by a lower sensitivity to empirical parameters.

We can also speculate that a higher sensitivity to the empirical parameter indicates that the variability from the physical part of the model is small, implying a lesser impact from the physical model. This interpretation would be reasonable if the total standard deviation of ET is not small when GSI is small. We found that a small GSI does not necessarily correspond to a small variability of estimated ET. In fact, the standard deviation of estimated ET is negatively correlated with GSI, although this relationship is weak and is not statistically significant (*R = −0.7, p = 0.083*) (Fig 7b). A negative correlation implies that a model’s sensitivity to empirical parameters is not the sole determinant of its overall variability. Even if a model has low sensitivity to these parameters, it can still exhibit high variability due to significant contributions from the physical equations governing the process.

### 5.4 Model performance for extreme conditions

**Fig 8:**
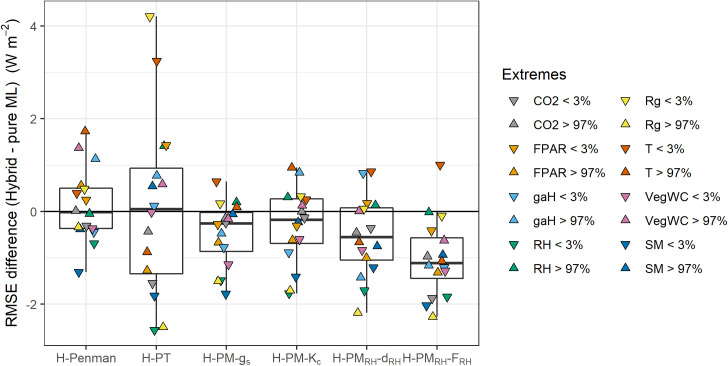
Model performance improvement using the hybrid approach for *LE* estimation under extreme environmental conditions. The performance improvement is quantified as differences in RMSE between the hybrid approaches and the Pure-ML model. Each boxplot represents the distribution of RMSE differences for a specific hybrid model compared to the Pure-ML model, across different environmental extremes. All input variables of the ML algorithm were used here, represented by different colors’ jitter points. Extremes are defined as lower (< 3%: downward pointing triangles) and higher (> 97%: upward pointing triangles) quantiles of test set data.

In this section, we evaluate how well each hybrid model improved ET estimation compared to the *Pure-ML* model under extreme conditions (Fig 8). The definition of extreme conditions can be found in section 3.3. The performance improvement varied significantly depending on the specific extreme conditions. However, when considering the median model improvement across all extremes tested, the *H-PM*_*RH*_*-F*_*RH*_ model generally shows consistently large performance improvements. Notably, this result remains consistent even when the definition of extreme conditions is adjusted to represent the 1% extreme values (upper or lower 1%), as well as upper or lower 2%, instead of 3%.

The *H-PT* model exhibits large performance degradation under low radiation conditions (*R*_*g*_ < 3%, that is, within the lowest 3 percentiles of observed *R*_*g*_ for each site) and low temperature conditions (*T* < 3%), resulting in a wide distribution of model performance in extreme conditions. This degradation is likely due to the PT equation’s structure, which doesn’t account for the aerodynamic term, particularly when temperature or radiation are low, and sensible heat advection is strong. Interestingly, the *H-PT* model displays the highest $\sigma_{m}/\sigma_{o}$ value overall (Fig 7b), yet this did not translate to model improvements under extreme conditions (Fig 8).

## 6 Discussion

### 6.1. Underlying reasons for the outperformance of PM_RH_ based models

Physically constrained hybrid approaches for estimating ET have evolved rapidly in recent years. While the specific implementations vary, most previous studies adopt one of the physical formulations used in our work in developing hybrid models [22–30]. These prior studies generally report comparable performance between pure ML and hybrid model, while maintaining consistency with physical constraints. This aligns with our findings, where all tested models showed relatively similar performance, while some hybrid models show moderate performance improvement.

A unique contribution of the present study lies in systematically evaluating multiple hybrid structures, which enabled us to explore how the integration of physical constraints can enhance model performance. Specifically, we found that hybrid models are more accurate when the intermediate parameter estimated by machine learning is both difficult to predict and less influential in propagating error into the final ET estimate. Notably, the two PM_RH_-based hybrid models demonstrated the lowest GSI values, leading to the lowest RMSE under both normal and extreme conditions. Here, we explore the theoretical reasons for this finding.

Salvucci and Gentine [98] showed that vertical gradients of relative humidity tend to be stable due to land-atmosphere equilibration, resulting in minimized variance of the relative humidity gradients. Later studies showed that the atmospheric boundary layer tends to stabilize atmospheric relative humidity over multi-day timescales, resulting in stable and minimal vertical relative humidity flux [93,99]. Indeed, previous studies found that setting *F*_*RH*_ to zero can approximate ET without considering the variability of *F*_*RH*_, which is known as Surface Flux Equilibrium (SFE) [100,101]. This implies that the first term on the right-hand side of Eqs. 7 and 8 (representing the SFE-based ET) already approximates actual ET reasonably well, unlike other physical formulations such as the PM potential ET, which deviate more substantially from actual ET.

As a result, reducing the residual error using machine learning within the PMRH-based hybrid model may be more effective than with other approaches. Furthermore, *F*_*RH*_ and *d*_*RH*_ exhibit lower variability than other empirical parameters due to the stabilizing influence of atmospheric boundary layer processes, leading to less error propagation in ET estimates (Fig 7). In contrast, the widely used *g*_*s*_ is known to be a major source of error in ET estimates due to its higher variability [102].

**Fig 9:**
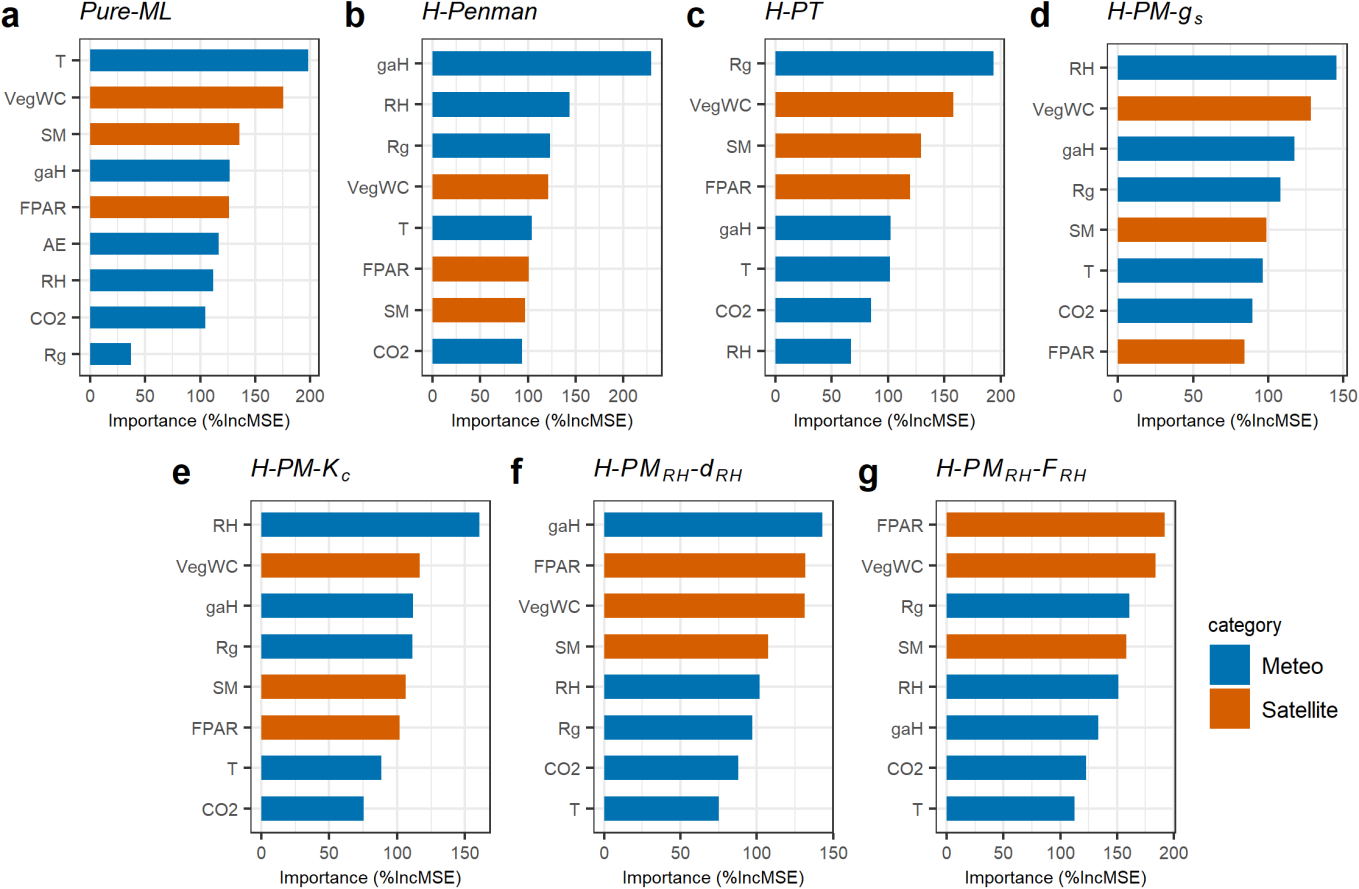
Variable importance in estimating *LE* using the *Pure-ML* (a), and variable importance in estimating each empirical parameter in the six hybrid models (b-g). Here, %IncMSE indicates the increase of the mean squared error when variables are randomly permuted. In-situ meteorological measurements are shown in blue (Meteo), and satellite-derived land surface parameters are shown in orange (Satellite).

The improved performance of *H-PM*_*RH*_*-F*_*RH*_ over *H-PM*_*RH*_*-d*_*RH*_ may be attributed to the uncertainty in *g*_*aH*_. In section 3.2, we detail how *g*_*aH*_ was estimated using a semi-empirical model, and explain why that introduces uncertainty as discussed in a previous study [26]. *H-PM*_*RH*_*-F*_*RH*_ model may reduce this uncertainty compared to *H-PM*_*RH*_*-d*_*RH*_ by embedding *g*_*aH*_ within the empirical parameter, $F_{RH}(=g_{aH}d_{RH})$. Additionally, negative feedback between *g*_*aH*_ and *d*_*RH*_ (e.g., hi*g*her *g*_*aH*_ corresponding to lower *d*_*RH*_ due to faster mixin*g*) results in more stable *F*_*RH*_ values compared to *d*_*RH*_. Variable importance analysis supports this feedback (Fig 9), showing that *g*_*aH*_ is the most important variable for predicting *d*_*RH*_ but not for *F*_*RH*_, even thou*g*h $g_{aH}$ is embedded in *F*_*RH*_.

Moreover, *H-PM*_*RH*_*-F*_*RH*_ is unique among the hybrid models in identifying satellite inputs as the most important variables (Fig 9). This suggests that meteorological data is effectively integrated into the physical equation, while the empirical parameter *F*_*RH*_ relies more on land surface information that is not included in the physical model.

### 6.2. Potential caveats

Despite the valuable insights it provides, our analysis is subject to several limitations. Firstly, we trained and evaluated our model using EC measurements, which incorporate systematic uncertainties due to the lack of energy balance closure. This means that the measured sum of latent and sensible heat fluxes is typically smaller than the measured *AE*, posing a challenge when employing physical models, as all the tested models assume energy balance closure. To address this issue, we utilized energy balance-corrected EC data based on the Bowen ratio preservation method [39,40]. However, it should be noted that this correction may result in an overestimation of ET [103].

Secondly, we utilized not only meteorological variables measured in the field but also satellite-driven data. However, due to the discrepancy between the footprint size representing EC observations and pixel size representing satellite remote sensing, remotely sensed variables may not accurately represent the land surface conditions observed by EC measurements, especially over heterogeneous land cover. For example, the SMAP soil moisture product has a spatial resolution of approximately 9 km, which is substantially larger than the typical EC footprint. As a result, the satellite-derived variables may not fully capture the local conditions observed by EC towers, potentially affecting model performance. Nonetheless, since all tested models, both hybrid and pure ML, used the same set of satellite inputs, the influence of this uncertainty on the machine-learned parameters is expected to be comparable across models. Therefore, the model comparisons remain valid despite this limitation. Moreover, satellite-driven information proved to be influential in determining empirical parameters in our variable importance analysis (Fig 9). This finding implies the suitability of utilizing satellite remote sensing as a predictor of site-level ET.

Third, in this study we employed only the RF algorithm to evaluate the performance of hybrid models relative to a pure ML model. Including additional machine learning algorithms would significantly increase the complexity of the model comparison. Therefore, we focused on RF, which has been shown to perform well in estimating ET compared to other ML algorithms [15]. Nonetheless, our findings remain subject to validation using alternative ML approaches such as artificial neural networks. Future studies could address this limitation by testing multiple ML algorithms within a simplified set of hybrid model configurations.

Finally, although we explore performance differences among the hybrid models and the *Pure-ML* model, the disparities in overall model performance were relatively small particularly in water units. This could be attributed to the ML model already performing at close to the measurement uncertainty [104]. Nevertheless, even these small differences in daily timescale errors can have a significant impact when a model predicts ET during extreme events, or when models are applied outside the current climatic ranges. Also, this accuracy enhancement is substantial compared to the hyperparameter tuning of RF model.

## 7 Conclusions

In this study, we evaluated physically-constrained hybrid ET models to enhance our understanding of how hybrid models improve ET estimation performance over pure ML approaches. Our key findings include: (1) employing hybrid models significantly enhances performance over the pure ML model, especially when compared to the gains from hyperparameter tuning of the RF algorithm; (2) the best-performing hybrid model, *H-PM*_*RH*_*-F*_*RH*_, consistently demonstrates high performance across the training/validation set, test set, various land cover types, and under extreme conditions; (3) generally, the more challenging it is to estimate the intermediate parameter requiring ML, the more accurate is the final ET prediction; and (4) minimizing the sensitivity of ET estimates to ML-determined parameters is crucial, as it reduces error propagation and leads to more robust performance improvements.

Our evaluation of six hybrid ET models in both normal and extreme conditions demonstrated that using vertical relative humidity flux as an ML-determined parameter exhibited the best performance compared to conventional approaches. This superior performance can be attributed to the relatively small variability of relative humidity flux, likely due to the Surface Flux Equilibrium theory and relatively lesser impact from uncertainty introduced by the semi-empirical aerodynamic conductance equation. This highlights the critical importance of domain knowledge in selecting appropriate physical models and parameter combinations for hybrid model development. Also, our results challenge the utility of the widely-used conventional parameters in hybrid approaches, as these conventional parameters, such as surface conductance, may not be the best option for hybrid methods due to their high variability and sensitivity.

While our study focused on ET, these insights are likely applicable to hybrid models for other quantities that require empirical parameters, even when physical processes are well defined. By leveraging domain knowledge, researchers can select better physical structures and parameters, ultimately enhancing the performance and reliability of hybrid models across various applications. For instance, similar approaches could be beneficial in modeling other hydrological or atmospheric processes.
